# Beware of AI programs bearing false gifts

**DOI:** 10.1093/jhps/hnad013

**Published:** 2023-05-27

**Authors:** Richard E Field

**Affiliations:** Editor-in-Chief, Journal of Hip Preservation Surgery, Oxford University Press

Human desire to communicate predates the written language by some 60 millennia [1]. As a small child, I was given crayons and taught to form the letters of the alphabet. As my dexterity improved, I progressed to an HB-grade Staedtler Tradition® 110 graphite pencil [2]. The pleasure of using a pencil sharpener has never diminished. In my teenage years, I wrote letters with my trusty, Parker 17, fountain pen. As a young doctor, I spent a week printing three copies of my thesis on a daisy-wheel printer [3], patiently feeding in sheets of 100gsm paper and changing the printer ribbon spools at the first sign of fading text. For the past 20 years, my keyboard and Google have guided me to sources of information, and emails have connected me to thousands of people across the world. Now we have Artificial Intelligence (AI) algorithms. AI knows me better than any of my friends and can predict my behaviour, perhaps better than my wife. Over the Christmas holiday, my children introduced me to ChatGPT [4]. They tell me that this will change my life.

Ever eager to embrace the new technology, I have asked ChatGPT to generate information sheets on indications, benefits and potential complications of various surgical procedures. I rapidly learned that the output evolved with iterative refinement of my questions. However, I remain reticent to share such information with my patients for fear that I may have crafted the wrong question and precluded content that would be relevant to their needs. I understand that AI researchers are developing patient-specific risk profiling [5]; however, such tools need to be robust before we can use them with confidence and know that the material is pitched at the appropriate level for patients of differing educational development and personality.

I have asked ChatGPT to generate systematic reviews, using ‘Preferred Reporting Items for Systematic Reviews and Meta-Analyses’ (PRISMA) methodology **[**6]. I have specified the databases to be explored, the keywords to be sought and the culling criteria and requested both clinical and radiological outputs. While ChatGPT can list references that appear to satisfy my nuanced search criteria, I can see that my final output still contains inappropriate information and I have no way of knowing what relevant material has been excluded. At present, the value of our systematic reviews depends on the background knowledge and powers of discrimination of human researchers to identify the most relevant literature. We need studies to identify whether ChatGPT and other AI tools can do this better.

I have asked ChatGPT how AI is being used in hip preservation surgery. My question was:

‘List ten ways in which AI can be used to support hip preservation surgery and list the most cited literature to support each example, provide the relevant references and number of citations. Exclude any work related to joint arthroplasty’. I ran the same query multiple times and, on each occasion, I received a different answer. On each occasion ChatGTP provided an impressive array of AI applications. These addressed topics such as planning and simulation of surgical procedures, image analysis and diagnosis, outcome prediction, surgical navigation, risk assessment and patient selection, rehabilitation and physical therapy, instrument tracking and optimization, data analysis and research, virtual coaching and education and patient communication. For each topic, I was provided a brief explanation of the research and a citation detailing the authors, journal, volume, issue and pages of the paper and also a citation count for the publication.

All but one of the journals were real but not one had published an article by the listed authors or a paper with the cited title. All were synthetic, imaginary publications.

In Issue 10.1, we can see how human scrutiny of world literature enables us to better understand the evolution of scientific research on a new surgical innovation. The paper by Peng et al. plots the rise in publications on surgical dislocation of the hip over the last two decades [7]. Rather than an AI search engine, the authors have used bibliometric analysis [8]. They have shown how a group pioneering a new technique spawn global research, where it is undertaken and the rate at which research is generated. I have not asked ChatGPT to replicate this study.

Another human strategy for the evaluation of opinion and research is achieved by bringing together experts and seeking consensus. In Issue 10.1, we publish the article titled ‘The 2022 International Society for Hip Preservation (ISHA) physiotherapy agreement on assessment and treatment of greater trochanteric pain syndrome (GTPS): an international consensus statement’ [9]. There is a search engine called ‘consensus.ai’ but it does not seem to be active at the moment. I did find an App called ‘Consensus’, which claims to provide ‘evidence-based answers faster’ [10]. However, when I asked it for guidance on greater trochanteric pain syndrome, it just told me a bit about the problem and listed a number of research studies on the subject. Google offered me 615 000 results on the subject. While I cannot argue that our consensus group have provided the definitive answer, AI does not yet offer a better alternative, and I commend this paper as a helpful appraisal of the subject by knowledgeable humans.

My third pick in Issue 10.1 is a case report by Kinsley and Safran titled ‘Medial approach for hip arthroscopy: a case report to access and treat osteoid osteoma of the medial femoral neck’ [11]. One of the challenges for any journal editor is to attract papers that will be read and cited by other researchers. In this regard, case reports are viewed as low value. This is a shame because case reports often provide the most interesting insights into real-world clinical practice and alert us to treatment options that we would not otherwise consider.

I hope that all our human-produced papers prove to be of interest to you all and we look forward to receiving AI-derived work as this technology matures.

## References

[1] Wikipedia. Cave painting. Available at: https://en.wikipedia.org/wiki/Cave_painting. Accessed: 15 March 2023.

[2] Staedtler. Graphite pencil. Available at: https://www.staedtler.com/intl/en/products/pencils-and-accessories/graphite-pencils/tradition-110-graphite-pencil-m110/. Accessed: 15 March 2023.

[3] Wikepedia. Daisy wheel printing. Available at: https://en.wikipedia.org/wiki/Daisy_wheel_printing. Accessed: 15 March 2023.

[4] ChatGPT GPT-3. GPT Chatbot: Advanced AI Chat. Available at: https://chat-gpt.org. Accessed: 15 March 2023.

[5] Penny-Dimri JC, Bergmeir C, Reid CM et al. Machine learning algorithms for predicting and risk profiling of cardiac surgery-associated acute kidney injury. *Semin Thorac Cardiovasc Surg* 2021; 33: 735–45. 32979479 10.1053/j.semtcvs.2020.09.028

[6] Prisma. Transparent reporting of systematic reviews and meta-analyses. Available at: http://www.prisma-statement.org. Accessed: 15 March 2023.

[7] Peng P, Wei T, Fang W et al. A bibliometric analysis and visualization of research trends on surgical hip dislocation. *J Hip Preserv Surg* 2022; 10: hnac049. 10.1093/jhps/hnac049 PMC10234387 37275829

[8] Cooper ID . Bibliometrics basics. *J Med Libr Assoc* 2015; 103: 217–8. 26512226 10.3163/1536-5050.103.4.013 PMC4613387

[9] Disantis A, Andrade AJ, Baillou A et al. The 2022 International Society for Hip Preservation (ISHA) physiotherapy agreement on assessment and treatment of greater trochanteric pain syndrome (GTPS): an international consensus statement. *J Hip Preserv Surg* 2023; 10: hnac050. 10.1093/jhps/hnac050 PMC10234389 37275836

[10] Consensus. Evidence-Based Answers, Faster. Available at: https://consensus.app. Accessed: 2 April 2023.

[11] Pierre KJ, and Safran MR. Medial approach for hip arthroscopy: a case report to access and treat osteoid osteoma of the medial femoral neck. *J Hip Preserv Surg* 2023; 10: hnad003. 10.1093/jhps/hnad003 PMC10234386 37275835

